# Trends in taxonomy of Triatomini (Hemiptera, Reduviidae, Triatominae): reproductive compatibility reinforces the synonymization of *Meccus* Stål, 1859 with *Triatoma* Laporte, 1832

**DOI:** 10.1186/s13071-021-04847-7

**Published:** 2021-06-26

**Authors:** Natália Regina Cesaretto, Jader de Oliveira, Amanda Ravazi, Fernanda Fernandez Madeira, Yago Visinho dos Reis, Ana Beatriz Bortolozo de Oliveira, Roberto Dezan Vicente, Daniel Cesaretto Cristal, Cleber Galvão, Maria Tercília Vilela de Azeredo-Oliveira, João Aristeu da Rosa, Kaio Cesar Chaboli Alevi

**Affiliations:** 1 Universidade Estadual Paulista “Júlio de Mesquita Filho” (UNESP), Instituto de Biociências , Rua Dr. Antônio Celso Wagner Zanin, 250, Distrito de Rubião Júnior , Botucatu, SP 18618-689 Brazil; 2 Laboratório de Entomologia em Saúde Pública, Departamento de Epidemiologia, Faculdade de Saúde Pública , Universidade de São Paulo, Av. Dr. Arnaldo 715, São Paulo, SP Brazil; 3 Laboratório de Parasitologia , Universidade Estadual Paulista “Júlio de Mesquita Filho” (UNESP), Faculdade de Ciências Farmacêuticas , Rodovia Araraquara-Jaú km 1, Araraquara, SP 14801-902 Brazil; 4 Laboratório de Biologia Celular , Universidade Estadual Paulista “Júlio de Mesquita Filho” (UNESP), Instituto de Biociências, Letras e Ciências Exatas , Rua Cristóvão Colombo 2265 , São José Do Rio Preto, SP 15054-000 Brazil; 5 Laboratório Nacional e Internacional de Referência em Taxonomia de Triatomíneos, Instituto Oswaldo Cruz (FIOCRUZ) , Av. Brasil 4365, Pavilhão Rocha Lima, sala 505, Rio de Janeiro, RJ 21040-360 Brazil

**Keywords:** Chagas disease vector, Triatomines, *T. longipennis*, *T. mopan*, Experimental crosses

## Abstract

**Background:**

*Meccus*' taxonomy has been quite complex since the first species of this genus was described by Burmeister in 1835 as *Conorhinus phyllosoma*. In 1859 the species was transferred to the genus *Meccus* and in 1930 to *Triatoma*. However, in the twentieth century, the *Meccus* genus was revalidated (alteration corroborated by molecular studies) and, in the twenty-first century, through a comprehensive study including more sophisticated phylogenetic reconstruction methods, *Meccus* was again synonymous with *Triatoma*. Events of natural hybridization with production of fertile offspring have already been reported among sympatric species of the *T. phyllosoma* subcomplex, and experimental crosses demonstrated reproductive viability among practically all species of the *T. phyllosoma* subcomplex that were considered as belonging to the genus *Meccus*, as well as between these species and species of *Triatoma*. Based on the above, we carried out experimental crosses between *T. longipennis* (considered *M. longipennis* in some literature) and *T. mopan* (always considered as belonging to *Triatoma*) to evaluate the reproductive compatibility between species of the *T. phyllosoma* complex. In addition, we have grouped our results with information from the literature regarding crosses between species that were grouped in the genus *Meccus* with *Triatoma*, in order to discuss the importance of experimental crosses to confirm the generic reorganization of species.

**Results:**

The crosses between *T. mopan* female and *T. longipennis* male resulted in viable offspring. The hatching of hybrids, even if only in one direction and/or at low frequency, demonstrates reproductive compatibility and homeology between the genomes of the parents.

**Conclusion:**

Considering that intergeneric crosses usually do not result in viable offspring in Triatominae, the reproductive compatibility observed between the *T. phyllosoma* subcomplex species considered in the *Meccus* genus with species of the *Triatoma* genus shows that there is “intergeneric” genomic compatibility, which corroborates the generic reorganization of *Meccus* in *Triatoma*.

**Graphic Abstract:**

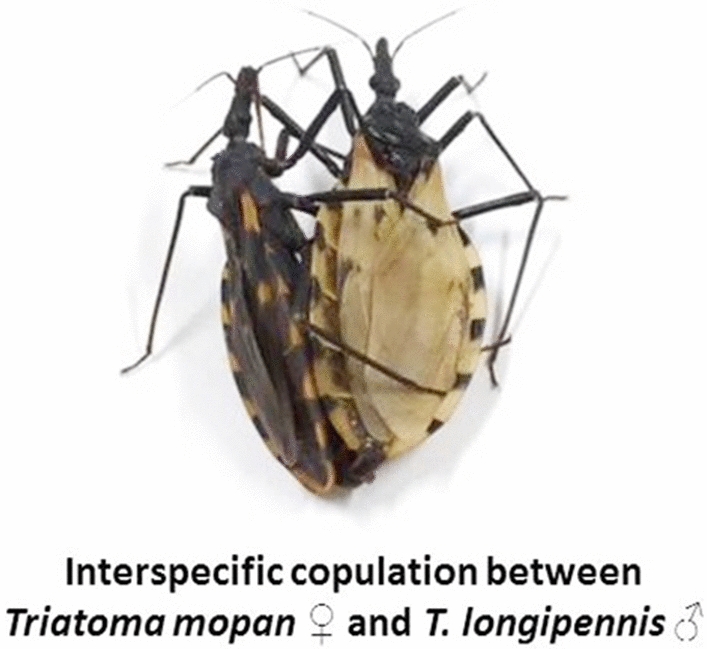

## Background

Triatomines are hematophagous insects of great importance for public health, since they are considered the main form of transmission of the protozoan *Trypanosoma cruzi* (Chagas, 1909) (Kinetoplastida, Trypanosomatidae), the etiological agent of Chagas disease [1]. Currently, there are 8 million infected people worldwide and around 25 million living in an area at risk of infection [1], the control of vector populations being the main measure for the reduction of new chagasic patients [1].

Triatomines are part of the Hemiptera order, Heteroptera suborder, Reduviidae family and Triatominae subfamily [2]. There are 156 species in this subfamily, distributed in 18 genera and five tribes [3–6]. The Triatomini tribe is composed of nine genera, namely, *Dipetalogaster* Usinger, 1939, *Eratyrus* Stål, 1859, *Hermanlentia* Jurberg & Galvão, 1997, *Linshcosteus* Distant, 1904, *Mepraia* Mazza, Gajardo & Jörg, 1940, *Nesotriatoma* Usinger, 1944, *Panstrongylus* Berg, 1879, *Paratriatoma* Barber, 1938, and *Triatoma* Laporte, 1832 [3, 4]. However, during the taxonomic history within this tribe, several genera have already been considered valid: *Eutriatoma* Pinto, 1926, *Conorhinus* Laporte, 1833*, Callotriatoma* Usinger, 1939*, Cenaeus* Pinto, 1925*, Neotriatoma* Pinto, 1931*, Lamus* Stål, 1859, *Mestor* Kirkaldy, 1904, *Triatomaptera* Neiva & Lent, 1940, and *Meccus* Stål, 1859 [7, 8]. *Eutriatoma*, *Conorhinus*, *Neotriatoma* and *Meccus* were the genera synonymous with *Triatoma* [7, 8].

*Meccus*’ taxonomy has been quite complex, since the first species of this genus was described by Burmeister [9] as *Conorhinus phyllosoma* Burmeister, 1835; in 1859 the species was transferred to the genus *Meccus* [10] and in 1930 to *Triatoma* [11]. However, in the twentieth century, Carcavallo et al. [12] proposed the revalidation of the *Meccus* genus based on morphological data (alteration corroborated by Hypsa et al. [13] through molecular studies). Finally, in the twenty-first century, Justi et al. [8], through a comprehensive study including more sophisticated phylogenetic reconstruction methods, again synonymized *Meccus* with *Triatoma*.

The six species initially considered as *Meccus* [*T. bassolsae* Aguilar, Torres, Jiménez, Jurberg, Galvão & Carcavallo, 1999, *T. longipennis* Usinger, 1939, *T. mazzottii* Usinger, 1941, *T. pallidipennis* Stål, 1872, *T. phyllosomus* (Burmeister, 1835), and *T. picturatus* Usinger, 1939], together with *T. bolivari* Carcavallo, Martínez & Pelaez, 1987, *T. mexicana* (Herrich-Schaeffer, 1848) and *T. ryckmani* Zeledón & Ponce, 1972, form the *T. phyllosoma* subcomplex [3]. This subcomplex, together with the *T. dimidiata* subcomplex [*T. dimidiata* (Latreille, 1811), *T. hegneri* Mazzotti, 1940, *T. huehuetenanguensis* Lima-Cordón et al., 2019, *T. mopan* Dorn et al., 2018, *T. brailovskyi* Martínez, Carcavallo & Pelaez, 1984, and *T. gomeznunezi* Martínez, Carcavallo & Jurberg, 1994], form the *T. phyllosoma* complex [3, 14, 15].

Events of natural hybridization with production of fertile offspring have already been reported among sympatric species of the *T. phyllosoma* subcomplex [16]. Experimental crosses demonstrated reproductive viability among practically all species of the *T. phyllosoma* subcomplex that were considered as belonging to genus *Meccus* in some literature [17, 18]. In addition, experimental crosses between these species and species of *Triatoma* from the *T. phyllosoma* subcomplex (*T. mexicana*) and the *T. lecticularia* complex [*T. recurva* (Stål, 1868)] also resulted in the production of hybrids [19, 20].

The study of reproductive barriers by experimental crossings was used in taxonomy (with emphasis on description, revalidation, and synonymization of species [5, 21, 22]) and systematics (with emphasis on the evolutionary relationship between species [23]) of Triatominae. Based on the above, we carried out experimental crosses between *Triatoma* species of the *T. phyllosoma* (*T. longipennis*) and *T. dimidiata* (*T. mopan*) subcomplexes, to evaluate the reproductive compatibility between species of the *T. phyllosoma* complex. In addition, we have grouped our results with information from the literature regarding crosses between species that were initially grouped in the genus *Meccus* with *Triatoma*, in order to discuss the importance of experimental crosses to confirm the generic reorganization of species.

## Methods

Reciprocal experimental crosses were conducted between *T. longipennis* and *T. mopan*. These two species were chosen because both belong to the *T. phyllosoma* complex [3, 14, 15], and *T. mopan* has never been considered as belonging to *Meccus*, unlike *T. longipennis*. The insects used in the experiment came from colonies kept in the Triatominae insectary of the School of Pharmaceutical Sciences, São Paulo State University (UNESP), Araraquara, São Paulo, Brazil. The experimental crosses were conducted in the Triatominae insectary, according to the experiments of Correia et al. [24] and Mendonça et al. [25]: the insects were sexed as fifth instar nymphs, and males and females were kept separately until they reached the adult stage to guarantee the virginity of the insects used in the crosses. For the experimental crosses, three couples from each set were placed in plastic jars (diameter 5 cm × height 10 cm) and kept at room temperature.

## Results and discussion

As observed for the crosses between *T. recurva* and *T. phyllosoma* (as *M. phyllosomus*) [20] and between *T. mexicana* and *T. longipennis* [19], only the direction between *T. mopan* female and *T. longipennis* male resulted in viable offspring (Table 1). The hatching of hybrids, even if only in one direction and/or at low frequency (Table 1), demonstrates reproductive compatibility and homeology between the genomes of the parents.

**Table 1:** Experimental crosses performed between *Triatoma* spp. and *Meccus* spp.

Crossing experiments					Number of eggs	Egg fertility
♀	*T. mopan*	x	*T. longipennis*	♂	161	98 (61%)
♀	*T. mazzottii*	x	*T. mexicana*	♂	18^a^	12^a^ (67%)
♀	*T. mexicana*	x	*T. mazzottii*	♂	14^a^	09^a^ (64%)
♀	*T. picturatus*	x	*T. mexicana*	♂	25^a^	19^a^ (76%)
♀	*T. mexicana*	x	*T. picturatus*	♂	32^a^	23^a^ (72%)
♀	*T. mexicana*	x	*T. longipennis*	♂	14^a^	9^a^ (64%)
♀	*T. phyllosomus*	x	*T. mexicana*	♂	208^a^	156^a^ (75%)
♀	*T. mexicana*	x	*T. phyllosomus*	♂	392^a^	295 (75%)
♀	*T. recurva*	x	*T. longipennis*	♂	71.0 ± 78.3^b^	6.0 ± 0^b^ (8,4%)
♀	*T. longipennis*	x	*T. recurva*	♂	74.8 ± 44.6^b^	6.0 ± 0^b^ (8%)
♀	*T. recurva*	x	*T. picturatus*	♂	94.8 ± 39.6^b^	5.7 ± 6.4^b^ (6%)
♀	*T. picturatus*	x	*T. recurva*	♂	136.0 ± 68.9^b^	12.3 ± 15.4^b^ (8.8%)
♀	*T. recurva*	x	*T. pallidipennis*	♂	91.2 ± 77.3^b^	5.0 ± 0^b^ (5.5%)
♀	*T. pallidipennis*	x	*T. recurva*	♂	54.0 ± 59.9^b^	14.5 ± 13.4^b^ (26.8%)
♀	*T. recurva*	x	*T. mazzottii*	♂	92.7 ± 56.5^b^	3.0 ± 1.3^b^ (3.2%)
♀	*T. mazzottii*	x	*T. recurva*	♂	119.8 ± 38.3^b^	5.3 ± 0.6^b^ (4.4%)
♀	*T. recurva*	x	*T. phyllosomus*	♂	127.8 ± 88.1^b^	26.0 ± 26.7^b^ (20%)

^a^ Martinez-Ibarra et al. [19]; ^b^ Martinez-Ibarra et al.[20]

Intergeneric crosses usually do not result in viable offspring in Triatominae (possibly due to pre-zygotic barriers, such as genomic incompatibility), as noted for the crossings between *Panstrongylus* and *Triatoma*, *Panstrongylus* and *Nesotriatoma*, *Rhodnius* Stål, 1859 and *Psammolestes* Bergroth, 1911 (KCCA, personal communication) and *Rhodnius* and *Triatoma* [26]. The reproductive compatibility observed between the *T. phyllosoma* subcomplex species considered in the *Meccus* genus with species of the *Triatoma* genus (Table 1) shows that there is “intergeneric” genomic compatibility, which corroborates the regrouping of species in the same genus carried out by Justi et al. [8].

The genus *Triatoma* is a paraphyletic group comprising 82 species [3, 5, 8]. There are species of *Triatoma* related phylogenetically to the genera *Panstrongylus*, *Paratriatoma*, *Linshcosteus* and *Hermanlentia* [8], which justifies the paraphyly of the genus. The inclusion of the species of the genus *Meccus* in *Triatoma* rescues a discussion about the application of the morphological characteristics used for a long time as diagnoses for the genera of the subfamily Triatominae (as recently highlighted by Monteiro et al. [27]).

Taxonomy is a fundamental science for the entomo-epidemiology of Chagas disease, because correctly classifying triatomines can assist in the activity of vector control programs [28]. Even though since 2014 the generic status of the species grouped in *Meccus* has been changed to *Triatoma*, several authors continued publishing articles using the *Meccus* nomenclature as valid [20, 29–46] and, quite mistakenly, as *Triatoma* (*Meccus*) *pallidipennis* [47–49]—since *Meccus* after the genus *Triatoma* (between parentheses) represents a subgenus and, so far, there are no valid subgenera in the subfamily Triatominae.

## Conclusion

Thus, through reproductive compatibility, we confirm the generic reorganization of *Meccus* in *Triatoma* proposed by Justi et al. [8]. In addition, we highlight the importance of the correct classification of the species of the *T. phyllosoma* subcomplex into this genus to avoid future misunderstandings by the scientific community and vector control programs.

## Data Availability

The data supporting the conclusions of this article are included within the article.
